# The Image-to-Intervention Workflow in Orthopaedic Surgery: A Framework for Diagnostic Certainty, Quantitative Imaging, 3D Modeling, Biomechanical Validation, and Surgical Decision-Making

**DOI:** 10.3390/diagnostics16213467

**Published:** 2026-10-23

**Authors:** Florina Popa, Cosmin Alec Moldovan, Corina-Cecilia Manole-Pălivan, Cristina Stănescu, Răzvan Adam

**Affiliations:** 1 Integrated Laboratory of Surgical Techniques and Medical Imaging, Medical-Pharmaceutical Research Center, Clinical Surgical Department, Faculty of Medicine and Pharmacy, “Dunărea de Jos” University of Galați, Domnească Street No. 47, 800008 Galati, Romania; 2 Department of Medical-Surgical and Prophylactic Disciplines, Faculty of Medicine, Titu Maiorescu University, Bucharest 031593; General Surgery Ward, ‘Witting’ Clinical Hospital, 010243 Bucharest, Romania; 3 Clinical Medical Department, Faculty of Medicine and Pharmacy, “Dunărea de Jos” University of Galați, Domnească Street No. 47, 800008 Galati, Romania; 4 Integrated Laboratory of Microbiology, Immunology, Nutrition, and Genetics, Medical-Pharmaceutical Research Center, Department of Morphological and Functional Sciences, Faculty of Medicine and Pharmacy, “Dunărea de Jos” University of Galați, Domnească No. 47, 800008 Galati, Romania; 5 Department of Medical-Surgical and Prophylactic Disciplines, Faculty of Medicine, Titu Maiorescu University, Bucharest 031593, Romania; Department of Orthopedics and Traumatology, Elias Emergency University Hospital, 011461 Bucharest, Romania

**Keywords:** digital orthopaedics, 3D printing, patient-specific modeling, surgical planning, biomechanics, computational modeling, uncertainty, validation, digital twin, decision-making

## Abstract

**Background/Objectives:** Digital orthopedic technologies are often evaluated as isolated tools even though clinical use depends on a sequence of transformations from diagnosis to intervention. This literature-informed conceptual framework defines the Image-to-Intervention (I2I) pathway and distinguishes diagnostic adequacy, geometric validity, biomechanical validity, decision relevance, execution accuracy, and patient-level outcomes. **Methods:** The orthopedic and cross-domain literature on imaging, segmentation, 3D modeling, additive manufacturing, computational modeling, patient-specific instrumentation, navigation, and digital twins was purposively mapped to I2I stages. Study design and validation function were recorded separately; the review was a purposive conceptual synthesis rather than a systematic evidence appraisal; no formal risk-of-bias or certainty assessment was performed. **Results:** The framework uses an early eligibility checkpoint before resource-intensive modeling and a later model-informed decision checkpoint. Nine provisional uncertainty and failure domains identify measurement, transformation, model-form, decision, implementation, and feedback problems without treating them as equivalent errors. A multidimensional maturity profile replaces a universal ordinal ladder. Among 81 retained research sources, 9 were systematic reviews or meta-analyses, 5 were narrative or current-concepts reviews, and 7 were randomized controlled trials; the remainder comprised non-randomized comparative, observational, case-based, technical, cadaveric, synthetic-surrogate, and computational studies. Direct empirical stage mappings were non-exclusive and were most frequent for I2I-4 and I2I-5 (33 sources each); guidance applicability was counted separately. Because the bibliography was purposively assembled, these distributions describe only the cited corpus. **Conclusions:** I2I complements rather than replaces established guidance for diagnostic efficacy, surgical innovation, computational-model credibility, AI reporting, and medical 3D printing. Its taxonomy, maturity profile, and reporting domains remain provisional and require consensus development and independent reliability testing before clinical or regulatory use.

## 1. Introduction

Why Digital Orthopedics Needs an Information-Chain Model

**Figure 1:**
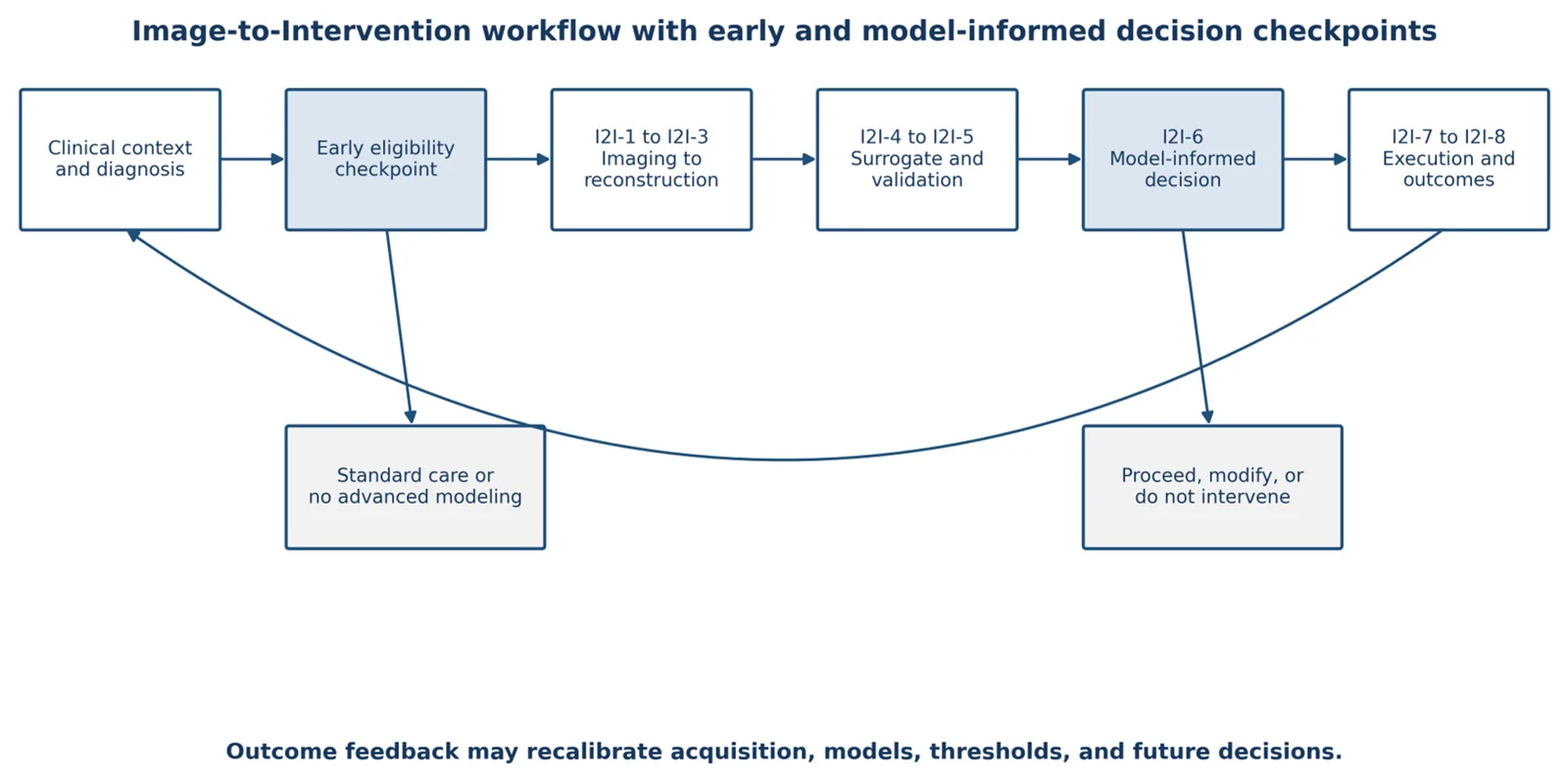
Revised Image-to-Intervention workflow. An early eligibility checkpoint precedes resource-intensive modeling, and a later model-informed decision checkpoint can lead to proceeding, modifying the plan, or non-intervention. Outcome feedback may recalibrate earlier stages.

Digital technologies are increasingly embedded in orthopedic surgery, but they are commonly discussed as separate innovations. Figure 1 organizes these technologies as a chain with two clinical checkpoints: an early determination that advanced modeling is appropriate and a later decision informed by the validated model. Each stage consumes information produced earlier and generates information that constrains the next stage; consequently, downstream performance cannot repair an invalid upstream premise.

Three-dimensional printing provides an intuitive example. A physical model may reproduce a digital mesh with submillimetric precision while the mesh itself is inaccurate because of segmentation error, and the segmentation may be internally reproducible while the source dataset lacks sufficient spatial resolution. Schulze et al. formalized several of these process-specific errors by separating segmentation error, digital-editing error, and printing error in patient-specific anatomical models [1]. The I2I framework extends this logic in both directions: upstream to diagnostic truth and image acquisition, and downstream to biomechanical validity, surgical indication, operative execution, and outcome feedback.

**Figure 2:**
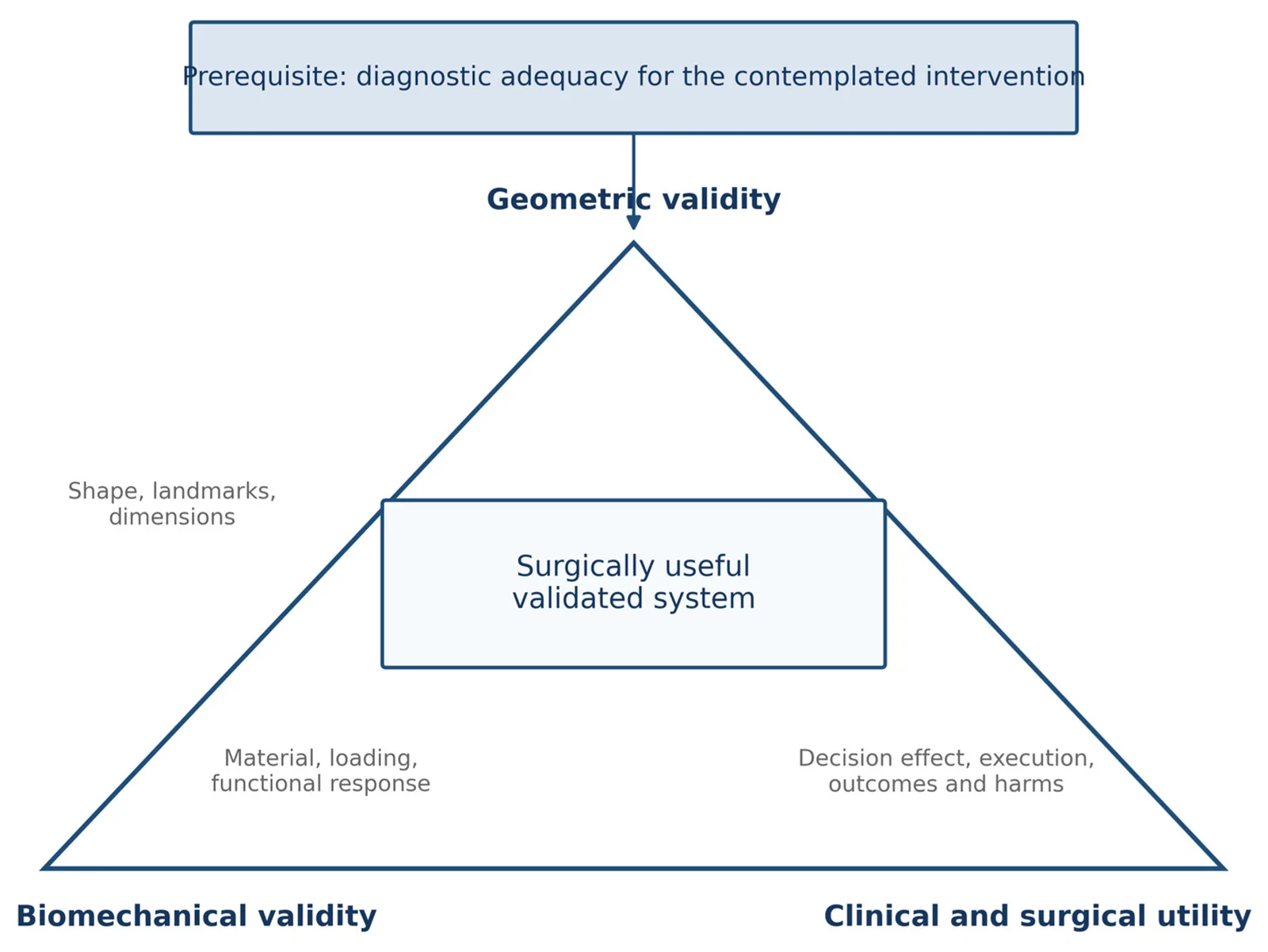
Validity architecture. Diagnostic adequacy is a prerequisite for the contemplated intervention. After that prerequisite is met, geometric validity, biomechanical validity, and clinical or surgical utility remain related but non-equivalent domains.

Diagnostic adequacy, geometric fidelity, biomechanical validity, and clinical utility are related but non-equivalent. Diagnostic adequacy is a prerequisite for applying the downstream validity domains shown in Figure 2. A printed bone may be geometrically accurate yet mechanically unrealistic, while a standardized synthetic model may be mechanically useful for comparing implants without reproducing biological heterogeneity [2,3,4]. The relevant question is therefore not whether a model is accurate in the abstract, but accurate for which decision and against which reference standard.

The same principle applies at the clinical end of the chain. Patient-specific surgical guides can reduce plan-to-result deviations in selected orthopedic procedures, but pooled accuracy varies by anatomical site and procedure [5]. Customized 3D-printed guides for ACL reconstruction have improved tunnel-positioning accuracy and reduced positioning time in recent comparative evidence, without necessarily producing measurable differences in short-term functional scores [6]. A technology can therefore improve execution accuracy without automatically improving every patient-centered outcome.

At an even more fundamental level, technological capability must not be confused with surgical indication. The historical debate surrounding arthroscopy for degenerative knee disease is a useful boundary case. The 2018 seed paper framed the concern as potentially unnecessary surgery [7], while a 2024 systematic review included ten randomized trials comparing arthroscopic surgery with conservative treatment for knee osteoarthritis; five trials contributed to meta-analyses, which found no significant advantage for pain, WOMAC function, SF-36, or satisfaction outcomes [8]. In the I2I model, this is not an exception but a required checkpoint: the chain must contain an indication gate at which the correct decision may be not to operate.

The purpose of this literature-informed conceptual framework paper is to define and critically examine an Image-to-Intervention pathway for orthopedic surgery. I2I links diagnostic adequacy, image acquisition, quantitative anatomy, reconstruction, physical or computational surrogates, validation, clinical decisions, execution, and outcome feedback. It is intended to expose dependencies and missing validation, not to replace established reporting, regulatory, or evaluation frameworks.

The manuscript also proposes a multidimensional maturity profile and provisional reporting domains. These tools are author-derived hypotheses: they are not a protocol, score, treatment algorithm, regulatory standard, or validated reporting statement. Table 1 positions I2I against established frameworks and identifies the narrower gap it is intended to fill.

### Positioning Against Existing Frameworks

I2I addresses a specific integration gap: existing frameworks usually evaluate imaging efficacy, innovation stage, computational credibility, AI reporting, manufacturing quality, or digital-twin foundations. I2I instead asks whether information remains fit for purpose as it crosses those boundaries and influences an orthopedic decision. Its novelty therefore lies in cross-stage traceability, not in replacing domain-specific standards [9,10,11,12,13,14,15,16,17,18].

**Table 1:** Positioning of I2I against established frameworks.

Comparator	Scope and Unit	Validation Domains	Decision and Outcomes	Added Role of I2I
Fryback–Thornbury [9]	Diagnostic imaging efficacy	Technical to societal efficacy	Explicit higher-level decision and outcome effects	Extends the chain beyond imaging into modeling and operative execution
IDEAL and IDEAL-D [10,11]	Surgical and device innovation over time	Development stage and study design	Clinical assessment and long-term follow-up	Maps information transformations within a patient-specific workflow
ASME V&V 40 and FDA guidance [12,13]	Computational-model credibility for a context of use	Verification, validation, uncertainty and risk	Credibility relative to a defined regulatory use	Connects computational credibility to diagnosis, surgical decisions and execution
CLAIM, TRIPOD+AI and DECIDE-AI [15,16,17]	AI imaging, prediction and early live clinical evaluation	Data, model, performance and implementation reporting	Decision-support evaluation and human factors	Covers non-AI and mixed physical-computational orthopedic chains
RSNA 3D-printing guidance [18]	Image-based anatomic models and clinical appropriateness	Acquisition, segmentation, manufacturing and QA	Appropriate clinical use of printed models	Links printing to mechanics, operative transfer and outcome feedback
National Academies digital-twin report [14]	Digital-twin foundations across domains	Model updating, uncertainty and infrastructure	Prospective prediction and lifecycle learning	Provides an orthopedic workflow-level application and gap map
I2I contribution	Patient-specific orthopedic information pathway	Diagnostic, geometric, mechanical, decision, execution and feedback validity	Two checkpoints plus plan-to-result and outcome linkage	A traceability crosswalk; not a replacement for the above frameworks

## 2. Materials and Methods

### 2.1. Review Design and Purpose

This article is a literature-informed conceptual framework paper rather than a systematic or scoping review. Its purpose is to integrate heterogeneous diagnostic, imaging, computational, biomechanical, and surgical evidence and to generate testable validation questions. It does not estimate a pooled treatment effect, claim exhaustive retrieval, or rank clinical interventions.

### 2.2. Information Sources and Search Approach

Literature identification used PubMed/MEDLINE, IEEE Xplore records, primary-publisher searches, and backward and forward citation tracking. No publication-date restriction was applied; the retained sources span 1986–2026. Searches were last updated on 12 August 2026. The concept blocks were (orthopaedic OR orthopedic) AND (CT OR MRI OR DICOM OR morphometry OR segmentation OR 3D reconstruction OR additive manufacturing OR patient-specific instrumentation OR finite element OR navigation OR robotics OR mixed reality OR digital twin) AND (accuracy OR reliability OR agreement OR validation OR decision OR outcome). Targeted combinations substituted the relevant anatomy or technology term. English-language human, cadaveric, synthetic-model, computational, review, guideline, and consensus sources were eligible when they informed at least one I2I stage, a validation endpoint, or a framework comparison.

Selection was purposive and author-led. Sources were excluded from the conceptual synthesis when they were duplicate records, lacked an identifiable validation or decision link, or repeated a claim already supported by a more direct source. The original draft did not retain a reproducible record-level log for the stated 110-candidate inventory. That numerical selection claim has therefore been removed rather than retrospectively reconstructed. No PRISMA flow diagram is presented.

The eight seed examples, identified in Supplementary Table S1, addressed degenerative arthroscopy, primary osseous Rosai–Dorfman disease, MRI-versus-CT reconstruction, acquisition-dependent knee modeling, ACL/notch morphometry, two comparative fifth-metatarsal surrogate-mechanics studies, and transfer of arthroscopic instruments to an extra-articular procedure. They were retained because they represented distinct prespecified boundaries in the original I2I concept. Author lists and reported affiliations were checked for all eight seeds. Reference [19] directly includes two current manuscript authors, Răzvan Adam and Cosmin Moldovan. The other seven—references [7,20,21,22,23,24,25]—include neither a current manuscript author nor an identified current-author institution, but they share an indirect co-authorship network through Mark Edward Pogărășteanu and/or Marius Moga; R.A. and C.M. coauthored reference [19] with Mark Edward Pogărășteanu. Accordingly, the seeds are disclosed as originating partly from an overlapping research network and are not treated as independent framework validation.

### 2.3. Evidence Characterization and Extraction

For each cited source, Supplementary Table S1 records a stable reference identifier, bibliographic identity, primary publication design, anatomical or technical domain, I2I stage role, validation endpoint, available sample-size or experimental-unit information, source-specific quantitative or qualitative finding, and key limitation. The fourth-round verification first matched DOI and title, then checked the original abstract and, when available, the full-text Methods and Results sections for publication design, population, intervention, sample or experimental unit, endpoint, principal finding, limitation, and stage assignment. For mixed-design publications, column F records the primary publication design; secondary analytical or validation functions are described in the endpoint, finding, and stage fields and do not change the publication-design count. Stage roles use explicit criteria: “Direct” means that the source measures or tests an endpoint assigned to that stage; “Direct synthesis” means that a review synthesizes such endpoints; “Contextual” means that the source is relevant to a stage without directly validating its endpoint; and “Guidance applicability” is counted separately from empirical evidence. Available numerical sample sizes were added; when an exact total was not provided in the accessible source or report, the entry states this explicitly. Reliability and agreement are also distinguished: reliability concerns discrimination among subjects or raters and is typically assessed with an intraclass correlation coefficient or kappa, whereas agreement concerns absolute difference and should use measures such as mean bias, limits of agreement, absolute error, or task-specific tolerance [26,27].

### 2.4. Framework Derivation and Appraisal Limits

The nine I2I stages were derived by decomposing the pathway from clinical context to outcome feedback into successive information transformations. The E0–E8 construct was then defined as provisional uncertainty and failure domains aligned with those transformations, and the maturity construct was revised into independent dimensions rather than a universal ordinal ladder. Existing frameworks were used as external comparators (Table 1). Because this work was designed as a purposive conceptual mapping exercise rather than a systematic review addressing a prespecified comparative-effectiveness question, no single formal risk-of-bias instrument or certainty-grading system was applied across the heterogeneous clinical, technical, cadaveric, computational, review, and guidance sources. Consequently, Supplementary Table S1 supports source-to-claim traceability but does not provide a graded estimate of evidentiary strength; the absence of formal appraisal limits confidence in the supporting evidence and precludes treating the mapped frequencies as proof of effectiveness. Comparative efficacy claims are therefore drawn preferentially from systematic reviews, randomized trials, guidelines, or consensus sources and are explicitly separated from illustrative or preclinical evidence.

### 2.5. Scope, Reproducibility, and Planned Validation

The taxonomy, maturity profile, and reporting domains were not developed by Delphi consensus and have not undergone independent inter-rater reliability testing. They are therefore presented as provisional constructs. The next validation phase should use at least two independent raters on a prespecified sample, report category-specific agreement and confidence intervals, revise ambiguous definitions, and perform formal content validation with multidisciplinary consensus. Supplementary Table S1, submitted as a separate workbook with this revision, provides the complete source-to-stage and source-to-claim map. Using stable reference identifiers, it separates the 81 research sources from the 14 framework, guidance, or methods sources (references [9,10,11,12,13,14,15,16,17,18,26,27,28,29]); reports primary publication design separately from validation function; distinguishes direct empirical mapping, contextual relevance, and guidance applicability; and records direct current-author overlap, reported institutional overlap, and indirect co-authorship-network relationships for all eight seed examples.

## 3. Results

### 3.1. Descriptive Profile of the Cited Evidence

The final bibliography contains 95 sources: 81 research sources and 14 methodological, reporting, regulatory, or framework sources. After source-level reclassification, the 81 research sources comprise 9 systematic reviews or meta-analyses, 5 narrative or current-concepts reviews, 7 randomized controlled clinical trials, 4 non-randomized comparative clinical studies, 10 observational cohort or cross-sectional studies, 7 case reports or descriptive case series, 16 technical or diagnostic validation studies, 6 cadaveric biomechanical validation studies, 8 synthetic or printed-surrogate biomechanical experiments, and 9 computational or engineering modeling validation studies. Mixed-design sources are counted once according to their primary publication design; their secondary validation functions remain documented separately in Supplementary Table S1. Direct empirical stage mapping uses the 81 research sources as its denominator and is non-exclusive: 2 sources directly address I2I-0, 12 I2I-1, 9 I2I-2, 25 I2I-3, 33 I2I-4, 33 I2I-5, 15 I2I-6, 22 I2I-7, and 18 I2I-8; 2 additional research sources are contextual-only. Guidance applicability uses the 14 framework, guidance, or methods sources as a separate denominator: 2 apply to I2I-0, 3 to I2I-1, 2 to I2I-2, 3 to I2I-3, 5 to I2I-4, 7 to I2I-5, 4 to I2I-6, 5 to I2I-7, 5 to I2I-8, and 3 are cross-cutting. These distributions are descriptive only of the purposively assembled bibliography and must not be interpreted as prevalence estimates for the wider literature. Available sample sizes or experimental units are now reported for the previously incomplete entries; where an exact number was not provided in the accessible source or report, this is stated explicitly. The audit corrected extracted details and increased the direct I2I-8 count from 17 to 18 after reference [30] was mapped to both execution and outcome/implementation endpoints, but it did not alter the proposed I2I stages, E0–E8 taxonomy, maturity-profile dimensions, or overall conceptual conclusions.

### 3.2. Diagnostic Adequacy as the Entry Requirement

The first stage of the I2I workflow precedes digital reconstruction. A model can be anatomically faithful and computationally precise while representing the wrong diagnosis. This problem is especially relevant in lesions with nonspecific radiological features, in which the treatment pathway depends on pathology rather than geometry alone.

Primary osseous Rosai–Dorfman disease provides an instructive boundary case. In the reported tibial lesion, imaging demonstrated an osteolytic process with cortical thinning, focal interruption, periosteal reaction, and later extraosseous extension, while initial pathological assessments were interpreted as chronic inflammatory tissue [19]. Definitive diagnosis required repeat excisional biopsy and immunohistochemistry. The case therefore demonstrates a type of upstream failure that no subsequent segmentation or 3D modeling can correct: if disease identity is uncertain, increased geometric precision can create false confidence rather than better decision-making.

For I2I purposes, diagnostic truth does not require that every condition be histologically proven. Rather, it requires that the level of diagnostic certainty be appropriate to the contemplated intervention. A traumatic fracture with characteristic imaging may have a very different evidentiary threshold from an indeterminate bone lesion. The proposed E0 error category therefore captures diagnostic uncertainty or misclassification before quantitative modeling begins. Studies describing patient-specific planning should report not only image-processing accuracy but also the clinical diagnostic context from which the model was derived. MRI performance in osteochondral disease likewise illustrates that diagnostic utility depends on the specific pathological question and classification task [31].

### 3.3. Image Acquisition and the Quality of Source Data

Image acquisition is the first explicitly digital stage. Orthopedic reconstruction pipelines commonly begin with CT or MRI DICOM data, but the modalities encode anatomy differently and are not interchangeable for every target structure. The 2018 MRI-versus-CT study described the conversion of DICOM data through segmentation to STL files and physical models, emphasizing CT’s favorable bone contrast and the potential value of MRI when soft tissue information or reduction in additional ionizing radiation is important [20].

The source dataset establishes an upper bound on downstream fidelity. Slice thickness, voxel dimensions, reconstruction kernel, signal-to-noise ratio, artifacts, field strength, and sequence choice affect the visibility of boundaries that later become segmentation surfaces. The seed knee-modeling study demonstrated this practically: an osteochondral lesion reconstructed from 0.8 mm spacing with 21 MRI images yielded materially different volume estimates from a dataset with 4.5 mm spacing and only four images [21]. The difference occurred before printing, illustrating E1-to-E2 error propagation.

MRI-based patient-specific modeling is increasingly feasible. Rankin et al. generated an ACL femoral tunnel guide from MRI and compared source anatomy, digital design, and the printed guide [32]. Black-bone MRI was developed in a dentomaxillofacial setting to facilitate bone-surface reconstruction without CT exposure [33]. Its citation here is therefore an explicit cross-domain analogy, not direct orthopedic clinical evidence. Such approaches shift rather than remove the need for acquisition and segmentation validation.

For the I2I framework, acquisition quality should be reported in relation to the final surgical task. A protocol adequate for gross fracture visualization may be inadequate for a millimeter-scale guide, osteochondral defect volumetry, or ligament-footprint morphometry. The relevant concept is therefore task-specific sufficiency rather than a universal minimum image resolution.

### 3.4. Quantitative Morphometry: Converting Anatomy into Surgical Data

Digital images become surgically actionable when anatomy is translated into reproducible measurements. Quantitative morphometry can provide patient-specific dimensions, areas, angular relationships, volumes, or shape descriptors that may influence reconstruction strategy.

The intercondylar-notch study illustrates this transition. MRI was used to quantify ACL area, anterior intercondylar-notch area, notch width, and related morphometric variables [22]. Different notch morphologies demonstrated different relationships between notch and native ACL dimensions. Importantly, the authors also acknowledged that measurement precision was constrained by 1.5-T MRI resolution and the exact overlap of axial sections with the notch, directly illustrating E2 measurement uncertainty.

The potential surgical value of morphometry lies not in classification for its own sake but in anticipating mismatch. In the same study, A- and W-shaped notches with smaller anterior notch dimensions were discussed in relation to a smaller native ACL and the possibility of graft impingement or notchplasty if graft dimensions exceed available space [22]. This is an archetypal I2I-2 to I2I-6 link: a measurement becomes relevant only when it changes the surgeon’s interpretation of anatomical constraint.

Modern morphometry is expanding from manually selected landmarks toward statistical shape modeling and machine-learning-assisted segmentation. These methods may improve throughput and allow population-level anatomical phenotyping, but they introduce additional sources of algorithmic and landmark uncertainty. Future I2I studies should therefore report measurement repeatability, observer dependence, model training/validation where AI is used, and the magnitude of error relative to the clinical decision threshold.

### 3.5. Segmentation, Reconstruction, and 3D Modeling

Segmentation is the transformation at which a grayscale or signal-intensity dataset becomes an explicit anatomical boundary. Thresholding, region growing, edge detection, manual contouring, semi-automatic methods, and increasingly deep-learning algorithms may all be used. The chosen method should be evaluated not only for visual plausibility but for reproducibility and task-specific error.

The MRI-versus-CT paper described the practical asymmetry between CT and MRI segmentation: CT bone segmentation can exploit relatively standardized attenuation values, whereas MRI lacks an equivalent universal intensity scale and may show overlap between adjacent tissues [20]. The knee-modeling study likewise found automated segmentation straightforward for a high-resolution CT dataset of normal knee bones, whereas defects often required manual segmentation [21].

The quality-assurance literature has formalized segmentation error as a major contributor to the final deviation of patient-specific anatomical models [1]. This is important because printing accuracy alone can be misleading. A printer may reproduce an incorrect mesh faithfully. The I2I framework therefore separates E3 segmentation/reconstruction error from E4 fabrication error and recommends reporting them independently whenever model accuracy is a study outcome.

The transition from segmented volume to STL or CAD representation adds further transformations: smoothing, decimation, hole filling, mesh repair, conversion between file formats, and design of guide or implant geometry. Each operation can alter the surface while appearing visually benign. The appropriate validation target depends on use. A teaching model may tolerate larger deviations than a cutting guide positioned near a neurovascular structure. Broader orthopedic reviews of patient-specific applications and guide production reinforce the need to preserve traceability across these transformations [34,35,36].

Early orthopedic studies established the clinical appeal of tangible fracture models [37] and virtual 3D reconstruction for complex tibial plateau planning [38]. Patient-specific guide studies then extended the pipeline from visualization to action, including osteotomy planning [39], ACL tunnel placement [32,40], and osteochondral allograft preparation [41]. The conceptual progression is therefore not simply from 2D to 3D, but from representation to intervention.

### 3.6. From Digital Geometry to Physical and Computational Surrogates

Once geometry has been reconstructed, it may remain virtual, be converted into a finite-element model, or be manufactured as a physical surrogate. These outputs answer different questions. A geometric model can improve spatial understanding; a finite-element model can estimate internal stress or strain under defined assumptions; a printed model can permit tactile planning, guide fabrication, or physical testing.

The two 2025 fifth-metatarsal studies illustrate the value of standardized printed surrogates for comparative mechanics [23,24]. In the Jones-fracture study, printed specimens reduced inter-specimen variability and provided a controlled environment for factorial comparison of T-plate and bicortical-screw fixation [23]. The same study explicitly cautioned that absolute failure loads may differ from cadaveric bone and characterized the models as reliable comparative tools rather than direct substitutes for biological bone. This distinction between comparative reproducibility and physiological fidelity is central to I2I-4.

Independent validation studies reinforce this point. Nägl et al. calibrated printed femoral models and showed that selected patient-specific printed constructs could reproduce aspects of strength, stiffness, and fracture behavior observed in human femora [2]. Metzner et al. performed biomechanical validation of additively manufactured artificial femoral bones [3], and Strand et al. found that some printed clavicle constructs were mechanically closer to elderly cadaveric clavicles than commercial synthetic models under several loading modes [4]. These findings support the use of additive-manufactured surrogates while also demonstrating that validity is material-, geometry-, and task-dependent.

The I2I framework therefore proposes that physical models be described according to the property they are intended to preserve. Geometric surrogates prioritize shape; educational surrogates prioritize spatial comprehension; biomechanical surrogates require material and structural calibration; surgical guides additionally require fit, sterilization compatibility, and plan-to-execution accuracy. A single label such as ‘3D-printed model’ obscures these different validation burdens.

### 3.7. Biomechanical Validation: Reproducibility Versus Physiological Fidelity

Biomechanical validation is the stage at which a model is asked to predict or compare behavior under load. It is also the stage at which visually convincing digital models are most vulnerable to overinterpretation. Mechanical validity depends on material constitutive behavior, geometry, contact definitions, boundary conditions, loading vectors, soft-tissue representation, and whether static or cyclic behavior is relevant.

The Jones-fracture experiment used controlled static loading and a factorial design to compare fixation constructs across load angles and interfragmentary distances [23]. The authors acknowledged that absence of cyclic loading, soft tissues, biological healing, and cadaveric heterogeneity limits direct clinical extrapolation. Those limitations do not invalidate comparative testing; they define the question the model can legitimately answer. Within a controlled surrogate, relative construct behavior may be informative even when absolute failure force is not physiologic.

The avulsion-fracture study increased experimental richness by combining multi-material 3D printing with digital image correlation and representative gait-phase loading [24]. DIC provides non-contact, full-field strain or displacement information rather than point measurements alone. The study used this capability to compare stress distribution between hook-plate and bicortical-screw constructs, while again acknowledging absent trabecular microstructure, anisotropy, soft tissues, and full physiological loading.

Digital image correlation has a broader validation role in biomechanics. Palanca et al. reviewed full-field displacement and strain measurement and highlighted dependence on speckle preparation, camera setup, correlation parameters, and operator decisions [42]. Matta et al. applied DIC to peri-implant bone strain [43]. Sánchez et al. optimized DIC parameters for engineering fatigue-crack analysis [44]; that source is used only to illustrate method-parameter sensitivity and is not presented as direct orthopedic clinical evidence.

The proposed E5 domain covers model-form and parameter uncertainty, including mismatches between a surrogate or simulation and the clinical mechanics it is intended to represent. Computational work should report code verification where applicable, mesh-convergence or discretization checks, material and boundary-condition sensitivity, calibration data, validation data kept distinct from calibration, and uncertainty relative to the context of use [12,13]. A model may be useful for ranking constructs even when it is not valid for absolute prediction, but that narrower claim must be explicit.

Table 2 demonstrates that the appropriate definition of validity depends on the type and intended function of the model. Imaging and morphometric data primarily require reproducibility and correspondence with anatomical ground truth, whereas digital meshes require validation against segmentation and surface or volumetric error. Anatomical 3D prints are principally concerned with dimensional fidelity, while printed biomechanical surrogates additionally require calibration of material and structural behavior against relevant experimental or cadaveric references. Computational models introduce further assumptions regarding constitutive properties, loading, contacts, and boundary conditions and therefore benefit from sensor-based, DIC, experimental, or in vivo validation. Patient-specific guides require direct plan-to-result assessment, whereas adaptive or digital-twin systems impose the highest validation burden because their predictions must remain reliable as longitudinal patient data are incorporated. Thus, model validity cannot be represented by a single universal accuracy metric; it must be defined according to the property that the model is intended to preserve and the decision it is intended to support.

### 3.8. Two Clinical Decision Checkpoints

An information chain should not proceed automatically from technical feasibility to intervention. I2I therefore uses two checkpoints. The early eligibility checkpoint occurs before resource-intensive imaging or model construction and asks whether the diagnosis, clinical question, expected value, burden, and available alternatives justify advanced modeling. The later model-informed checkpoint occurs after validation and asks whether the new information supports proceeding, modifying the plan, choosing an alternative, or not intervening.

Degenerative knee arthroscopy is a useful historical example because technical success does not establish durable clinical benefit. The 2018 contextual paper raised the question of potentially unnecessary surgery [7], while randomized trials and a later meta-analysis found no consistent clinically meaningful advantage of routine arthroscopy over non-operative care for degenerative knee disease or osteoarthritis [8,58,59]. Better imaging or visualization should therefore improve discrimination and shared decision-making rather than lower the intervention threshold.

The same distinction appears in patient-specific instrumentation. Customized guides may improve transfer of a preoperative plan, but a smaller plan-to-result deviation does not necessarily produce better patient-reported outcomes. In ACL reconstruction, MRI- or CT-derived patient-specific guides can improve tunnel-positioning accuracy and reduce positioning time [6,32,40,52], while the clinical relevance of small positional improvements depends on graft choice, tunnel biology, rehabilitation, and the magnitude of the baseline technical error. A randomized trial of patient-specific instrumentation in routine total knee arthroplasty likewise found no superiority over conventional instrumentation in patient-reported outcomes despite the theoretical promise of personalization [60].

Conversely, high-value indications occur when anatomy is sufficiently complex that conventional two-dimensional planning leaves substantial uncertainty. Studies of corrective osteotomy demonstrate accurate transfer of three-dimensional planning and, in several series, reductions in fluoroscopy or improved correction accuracy [30,39,61]. Patient-specific femoral osteotomy guides in total hip arthroplasty have achieved close agreement between planned and executed levels [53]. In oncological surgery, patient-specific guides may assist the translation of virtual resection planes to complex pelvic or periarticular anatomy, but oncological margin principles remain upstream constraints and cannot be subordinated to geometric convenience [62,63,64]. More recent pediatric orthopedic-oncology experience using in-house 3D-printed resection and graft-specific guides reported close agreement between planned and achieved resection length, while the small retrospective cohort and disease-specific context require cautious generalization [65].

The indication gate therefore separates two questions that are often conflated: can a technology perform a task accurately, and should the task be performed in this patient? The first is a technical-validation question; the second is a clinical-utility question. A mature I2I study should state both.

### 3.9. From Planning to Execution: Closing the Plan-to-Result Gap

Patient-specific planning becomes clinically meaningful only when the operative result can be compared with the plan. This plan-to-result comparison is the defining validation target of I2I-7. It converts a digital workflow from a visualization tool into a testable surgical system.

Early virtual planning studies demonstrated feasibility in complex fracture reconstruction and osteotomy [38,39]. More recent work has quantified the translation step directly. In tibial plateau fractures, postoperative CT can be registered to the preoperative virtual reduction to measure residual gap, step-off, and fragment translation [66]. This type of analysis is valuable because it distinguishes the quality of the virtual plan from the surgeon’s ability to reproduce it. A good virtual reduction followed by a poor postoperative match identifies an execution problem; an accurate execution of an inadequate plan identifies a decision or planning problem.

Patient-specific cutting and drilling guides are a physical method for encoding the plan into the operative field. Contemporary knee osteotomy series show that such guides can improve the fidelity of intended correction and reduce fluoroscopy, although clinical outcome differences may remain small or absent at short-term follow-up [30,61]. In ACL reconstruction, cadaveric and clinical studies have shown improved tunnel-placement accuracy using customized guides [32,40,52]. Similar principles extend to MPFL reconstruction, where randomized evidence suggests improved tunnel placement and earlier recovery with 3D-printed guide plates [67], and to total hip arthroplasty, where patient-specific femoral guides can reproduce planned osteotomy levels with millimetric accuracy [53].

Execution accuracy is not solely a property of the guide. It depends on the fit surface selected during design, surgical exposure, cartilage or soft-tissue preservation, seating of the guide, fixation stability, saw or drill clearance, registration, and operator experience. These human–device interactions are part of E7. A technically accurate guide may still fail if it is seated on a surface altered by osteophyte removal or if soft tissue prevents full contact. Conversely, a guide with moderate geometric error may still produce an acceptable clinical result if the decision threshold is broad.

Minimally invasive and endoscopic techniques introduce an additional transfer problem because visualization is constrained. The calcaneal-spur seed paper described the use of instruments developed for arthroscopy in an endoscopic extra-articular procedure and concluded that the ‘tools and methods developed in arthroscopic surgery can be extended’ to other orthopedic applications [25]. The I2I interpretation is that technology transfer should occur only after the target anatomy, access corridor, indication, and risk structures have been validated for the new use.

Navigation and intraoperative sensors can modify execution in real time, while postoperative imaging can quantify plan-to-result deviation. Navigation studies and sensor-assisted arthroplasty provide direct support for this statement [68,69]; alignment classifications and knee phenotypes [70,71] are relevant to planning targets but are not evidence that sensors modify execution. Mixed-reality visualization may improve interobserver fracture-classification reliability, although effects on operative outcomes remain unproven [72].

## 4. Discussion

### 4.1. Error Propagation Across the Image-to-Intervention Chain

The central proposition of the I2I framework is that precision and validity are not synonymous. A downstream step can be executed with extreme precision while faithfully preserving an upstream error. The clinically important unit of validation is therefore not the isolated technology but the chain.

E0 diagnostic uncertainty can be a consequential upstream failure, but the relative frequency and impact of E0 versus other domains have not been quantified. The Rosai–Dorfman case illustrates a conceptual possibility: accurate geometry could coexist with an unresolved diagnosis [19]. It does not establish that E0 is universally the most consequential domain.

E1 acquisition error determines what information is available for reconstruction. The MRI-versus-CT seed paper and subsequent CT/MRI comparison literature show that modality, slice thickness, voxel geometry, sequence, and image contrast influence the ease and accuracy of bone segmentation [20,33,45,73,74,75,76]. The effect may be subtle at gross scale but important when the final task involves a narrow tunnel, cartilage surface, osteotomy plane, or small implant.

E2 measurement error arises when anatomy is converted into quantitative variables. The ACL/notch study demonstrates that clinically interesting morphometric relationships remain constrained by image resolution, section selection, and observer reliability [22]. Independent studies confirm that notch and ACL dimensions and broader knee morphometry can be associated with injury patterns or risk [77,78]. The relevant error is not simply the numerical standard deviation; it is the probability that measurement uncertainty crosses a decision threshold.

E3 segmentation and reconstruction error has received more formal quality-assurance attention. Interobserver studies show that segmentation agreement varies by anatomical landmark and experience [1,46]. Automated algorithms can reduce time and improve reproducibility, but external validation remains essential. Current deep-learning systems range from task-specific robotic-TKA CT segmentation to foundation-model approaches capable of segmenting bone across MRI locations [47,79,80,81]. Fully automated output should therefore be considered a first-pass hypothesis unless performance has been demonstrated in the relevant anatomy and acquisition domain.

E4 fabrication and material-surrogate error becomes important when a digital model is converted into a physical object. A print may have excellent dimensional fidelity while using material properties that are poorly matched to human bone. Studies of femoral, clavicular, metatarsal, and trabecular surrogates show that additive manufacturing can reproduce selected mechanical properties, but the degree of fidelity depends on material, architecture, infill, orientation, and anatomy [2,3,4,82,83]. The two fifth-metatarsal seed studies correctly limit their strongest conclusions to comparative mechanics rather than direct prediction of in vivo failure [23,24].

E5 biomechanical-model error includes boundary conditions, loading vectors, contact definitions, tissue constitutive properties, muscle-force estimation, and calibration targets. Recent patient-specific FE and neuromusculoskeletal studies demonstrate that model output can change materially when geometry, activation, or loading assumptions change [49,50,51,57,84,85]. Validation against pressure sensors, instrumented implants, dynamic imaging, or full-field experimental measurements is therefore essential when simulations are used to influence treatment.

E6 indication error occurs when technically valid information is translated into an intervention without sufficient evidence that intervention is appropriate. Degenerative arthroscopy provides the clearest example [7,8,58,59]. E7 execution error is the deviation between the plan and the achieved operation, measurable through postoperative CT, radiographs, navigation logs, or guide-position analysis [5,30,52,53,61,66,67]. E8 feedback error is the failure to measure whether the planned anatomical or mechanical change was achieved and whether it improved outcomes.

**Figure 3:**
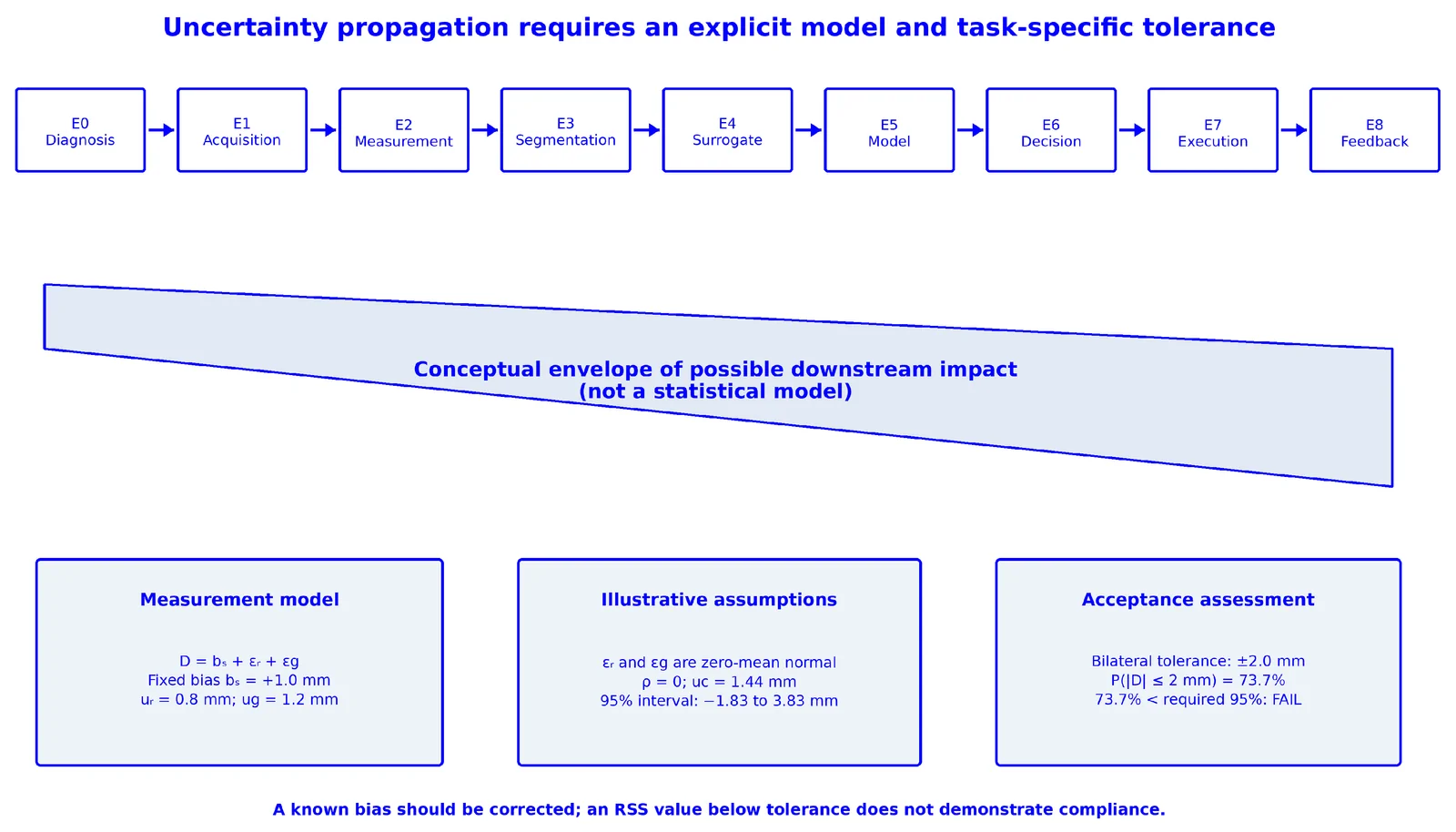
Conceptual uncertainty propagation and worked tolerance example. For the signed target-direction deviation D = b_s_ + εᵣ + εg, b_s_ is a fixed bias and εᵣ and εg are random components. The illustrative model fails a requirement that at least 95% of deviations fall within ±2 mm; an RSS value below the tolerance is not sufficient evidence of compliance.

E0–E8 are therefore described as uncertainty and failure domains rather than equivalent statistical errors. E1–E3 concern acquisition, measurement, and transformation uncertainty; E4–E5 concern surrogate and model-form validity; E6 is a decision failure; E7 is an implementation deviation; and E8 is missing or inadequate feedback. The domains may be dependent, non-additive, and differently consequential across tasks. Figure 3 is a conceptual warning. Its worked example defines a one-dimensional measurand and an explicit probabilistic acceptance rule; it does not imply universal error aggregation.

Worked example. Let D = b_s_ + εᵣ + εg denote signed target-direction deviation. The uncorrected segmentation contribution is a fixed bias b_s_ = +1.0 mm; independence of random terms does not randomize or remove that bias. Registration and guide-seating terms are modeled illustratively as zero-mean normal variables with standard uncertainties uᵣ = 0.8 mm and ug = 1.2 mm. With correlation ρ, the combined standard uncertainty is uc = (uᵣ^2^ + ug^2^ + 2ρuᵣug)^1^ᐟ^2^. Assuming ρ = 0 gives uc = 1.44 mm, not 1.75 mm, and a 95% interval around the biased mean of 1.0 ± 1.96 × 1.44 mm, or approximately −1.83 to 3.83 mm. For a bilateral tolerance of ±2.0 mm, P(|D| ≤ 2.0 mm) = 73.7%, which fails the prespecified acceptance criterion P(|D| ≤ 2.0 mm) ≥ 95%. A known bias should be corrected before compliance assessment; uncertainty in the bias estimate would then be propagated as a standard uncertainty. If normality or independence is not defensible, the joint distributions and correlations should be estimated and propagated analytically or by Monte Carlo simulation. Thus, an RSS magnitude below 2 mm does not by itself establish compliance.

The principal sources of uncertainty associated with each stage of the I2I pathway are synthesized in Table 3. The proposed E0–E8 taxonomy spans diagnostic misclassification, acquisition and resolution limitations, morphometric measurement error, segmentation and mesh-processing error, fabrication or material-surrogate limitations, modeling and loading assumptions, inappropriate clinical indication, execution or registration error, and failure to obtain meaningful outcome feedback. The corresponding mitigation strategies emphasize that uncertainty should be addressed at the stage where it originates: diagnostic discordance should be resolved before modeling; acquisition and morphometric limitations should be explicitly reported; segmentation and fabrication require method-specific quality assurance; biomechanical models require sensitivity analysis and experimental validation; and surgical translation requires both an evidence-based indication and plan-to-result assessment. Accordingly, the taxonomy is intended not simply to catalogue possible errors, but to make the provenance of downstream uncertainty visible and potentially correctable.

### 4.2. A Multidimensional I2I Maturity Profile

The term digital twin is increasingly used in musculoskeletal medicine, but patient specificity alone does not establish maturity. Established digital-twin work emphasizes model purpose, updating, uncertainty, verification, validation, and lifecycle governance [14]. Because orthopedic pathways differ, maturity should not be represented as a universal ladder in which every technology must pass through the same mechanical sequence.

The revised I2I profile therefore assesses six dimensions independently: geometric validity, mechanical or functional validity, decision relevance, execution accuracy, patient-level clinical outcomes, and adaptivity. Each dimension may be recorded as not assessed, internally validated, externally validated, or clinically or longitudinally validated. A dimension may be designated not applicable only when the intended use makes that decision defensible and the rationale is stated.

This structure accommodates heterogeneous pathways. A patient-specific guide may have strong geometric and execution validation without mechanical modeling. An AI decision-support tool may require external predictive and clinical evaluation without a physical surrogate. A biomechanical digital twin requires verification, calibration, sensitivity analysis, and longitudinal updating but may not include operative execution. These differences are not defects; they are reasons to display a profile rather than collapse maturity into one score [12,13,14,15,16,17,50,51,55,56,84,86].

Advancement in one dimension does not compensate for a missing critical dimension. Demonstrating that an output changes a decision establishes decision linkage, not decision quality. Likewise, plan-to-result accuracy and patient-level benefit remain distinct. The context of use determines which dimensions are critical and what evidence is sufficient [12,13].

**Figure 4:**
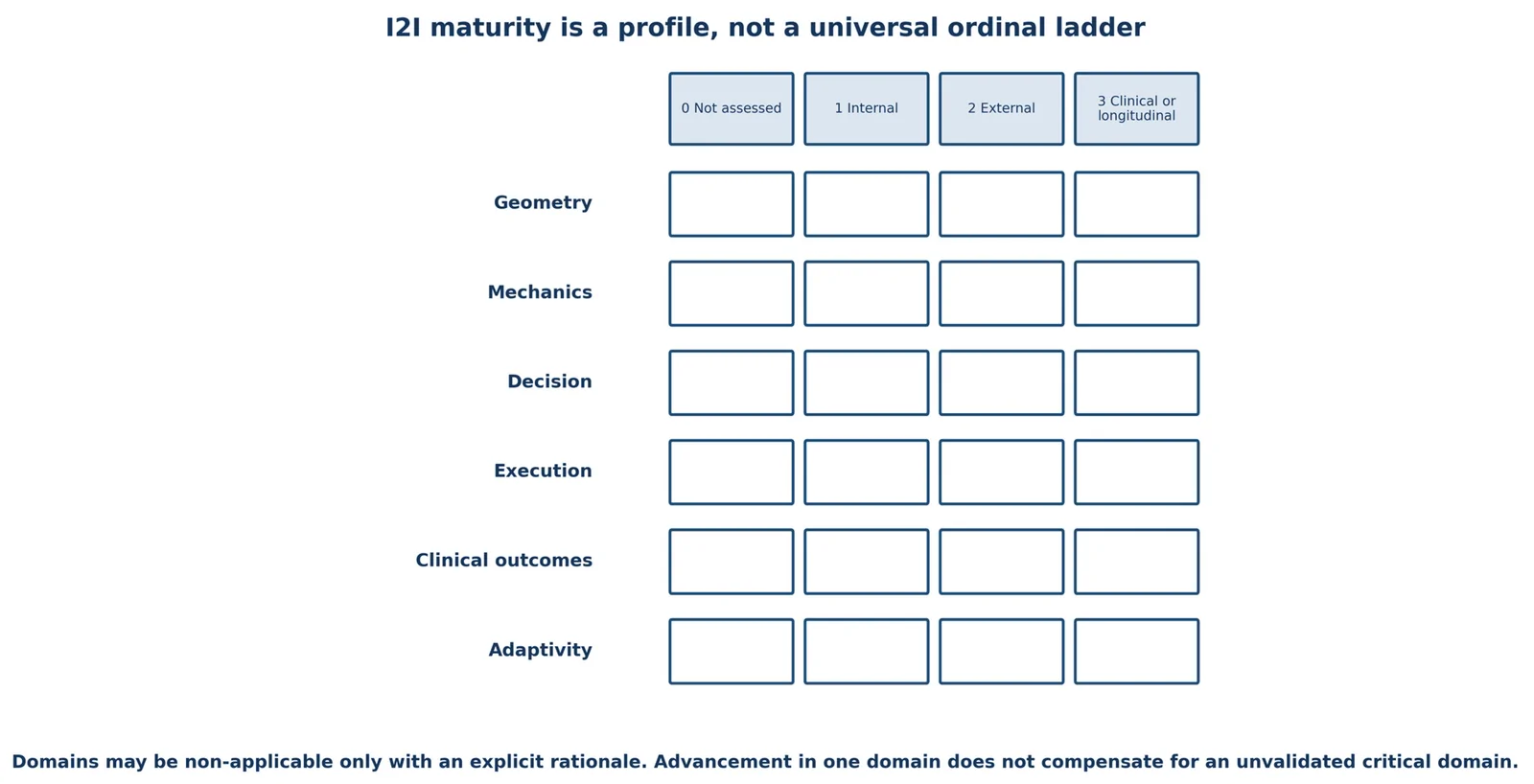
Provisional multidimensional I2I maturity profile. Geometry, mechanics, decision relevance, execution, clinical outcomes, and adaptivity are assessed separately from not assessed to internal, external, or clinical/longitudinal validation. Domains may be non-applicable only with an explicit rationale.

A mature adaptive orthopedic system would combine patient anatomy, tissue state, loading, symptoms, procedural history, and longitudinal outcomes; update predictions when new data arrive; monitor drift; and communicate uncertainty. Figure 4 summarizes the revised profile. It is provisional and requires content validation and inter-rater testing before use for formal classification.

### 4.3. Provisional Application of the Image-to-Intervention Framework

I2I can be used as a provisional documentation and research-mapping aid. It is not a validated checklist and should not be used as a gate that independently authorizes or prohibits an operation. Its function is to make evidence, assumptions, and missing validation visible alongside established clinical governance and regulatory requirements.

The nine stage-aligned questions are as follows: (1) Is the diagnosis sufficiently certain, and is advanced modeling appropriate for the clinical problem? (2) Does the source imaging contain the structures and resolution required for the task? (3) Which quantitative variables matter, and with what reliability and agreement? (4) How was anatomy segmented and reconstructed, and how was geometric error assessed? (5) Which properties must a physical or computational surrogate preserve? (6) How was the surrogate or simulation verified, calibrated, and validated for its context of use? (7) Does the information support an appropriate indication or technical choice compared with alternatives? (8) How was the plan transferred and how closely was it reproduced? (9) Did the achieved result improve anatomical, functional, patient-reported, safety, or resource outcomes, and can those data update the model?

A provisional I2I record, informally termed a passport, could store the answers with data provenance, software and version identifiers, known uncertainty, intended use, operator checks, and postoperative verification. This proposal has not undergone consensus development or reliability testing and should be regarded as a research concept rather than a reporting statement.

The present evidence demonstrates use across trauma, sports surgery, arthroplasty, oncology, hip, spine, and pediatric examples, but it remains weighted toward knee and 3D-printing applications [5,6,22,30,53,62,63,65,76,87,88]. Claims of anatomical generality have therefore been narrowed. Separate validation is needed for shoulder, spine, hip, pediatric, robotic, weight-bearing CT, metal-artifact, and 2D-to-3D workflows because their acquisition constraints, targets, and risks differ.

Importantly, I2I does not imply that every patient requires every stage. A simple fracture may proceed from diagnostic imaging directly to standard treatment. The framework becomes most valuable when complexity, personalization, or technological mediation increases. Its purpose is not to add steps but to make hidden assumptions visible.

As summarized in Table 4, the I2I framework is organized into nine successive stages, beginning with diagnostic truth (I2I-0) and progressing through image acquisition, quantitative anatomy, digital reconstruction, creation of physical or computational surrogates, validation, indication and decision-making, operative execution, and outcome feedback. Each stage is associated with a distinct validation question and a characteristic output. Importantly, progression through the framework does not imply that technical complexity necessarily increases at every step; rather, each stage requires confirmation that the information generated remains sufficiently valid for the next clinical or technical decision. The framework therefore shifts attention from the isolated performance of individual technologies toward the integrity of the complete information pathway, culminating in confirmation that the planned intervention has translated into an appropriate anatomical or patient-level outcome.

### 4.4. Research Gaps and Standardization Priorities

Several recurring gaps limit translation. First, validation is fragmented. Dice overlap alone is insufficient for a clinically relevant segmentation claim because it can conceal boundary displacement, small-structure error, or local outliers. Reports should combine overlap with HD95, average symmetric surface distance, surface Dice at a stated tolerance, absolute volume or landmark error, and task-specific target error [28]. Reliability and agreement must be labeled and analyzed separately [26,27].

Second, external validation remains insufficient for automated methods. A segmentation network can perform well on data acquired with the same scanner, sequence, population, and annotation protocol yet fail after domain shift. Recent foundation-model and external-validation studies are promising [47,79,80,81], but clinically relevant deployment requires multi-center, multi-vendor, multi-protocol testing and explicit failure analysis.

Third, the mechanical validity of printed surrogates remains task dependent. Controlled printed or synthetic models reduce biological variability and are attractive for comparative implant testing [2,3,4,23,24,82,83]. Separate cadaveric fifth-metatarsal experiments provide clinically grounded comparator mechanics [89,90,91,92], but they do not validate printed or synthetic surrogates unless paired with direct calibration under matched loading conditions. Reporting should include elastic properties, internal architecture, print orientation, manufacturing repeatability, loading rate, cyclic behavior when relevant, and the rationale for the chosen reference standard.

Fourth, computational models require verification and validation as well as uncertainty quantification. Reports should include code verification when relevant, mesh-convergence analysis, parameter and boundary-condition sensitivity, calibration procedures, independent validation data, and uncertainty relative to the stated context of use. Patient-specific geometry does not compensate for unverified numerics or population-average material and loading assumptions [12,13,51].

Fifth, technical accuracy must be linked to patient-centered utility. A guide that improves tunnel position, osteotomy angle, or femoral resection level is valuable, but the magnitude of the geometric improvement should be compared with functional outcomes, complications, reoperations, cost, radiation, operative time, and the minimal clinically important difference relevant to the procedure [30,52,53,60,61,67].

Sixth, the human factor is underrepresented. Segmentation depends on observer expertise [46]; guide placement depends on exposure and user familiarity; surgical decision aids depend on comprehension and trust. The clinician remains part of the I2I chain. Future studies should report training requirements, failure modes, override behavior, and inter-operator variation.

Finally, interoperability and traceability are prerequisites for mature systems. DICOM, segmentation masks, STL/STP meshes, CAD files, FE models, navigation data, sensor outputs, and patient-reported outcomes frequently reside in separate software ecosystems. Reproducible I2I research will require versioned data provenance and a record of every transformation capable of altering geometry or mechanics.

### 4.5. Cross-Cutting Implementation Domains

Technical validity is necessary but insufficient for implementation. Regulatory classification and intended use determine the evidence burden; point-of-care manufacturing requires a quality system, device traceability, controlled materials, post-processing, dimensional inspection, and validated sterilization and biocompatibility where patient contact occurs [18,29]. Deployment also requires assessment of cost-effectiveness, workflow time, data privacy, cybersecurity, algorithmic bias and domain shift, professional liability, training, override behavior, and human-factors engineering. These domains cut across I2I stages and can prevent an accurate system from reaching safe routine use.

### 4.6. Future Directions

#### 4.6.1. Uncertainty-Aware Artificial Intelligence

AI-assisted segmentation may become a common entry point for patient-specific reconstruction, but adoption will depend on external validation, failure detection, human review, and domain-shift monitoring rather than performance on one development dataset. CLAIM, TRIPOD+AI, and DECIDE-AI already define reporting expectations for medical-imaging AI, prediction models, and early live clinical evaluation; I2I adds cross-stage traceability and does not duplicate those documents [15,16,17].

#### 4.6.2. Multimodal and Low-Radiation Personalization

Improved MRI-based bone reconstruction, black-bone sequences, statistical shape modeling, and multimodal registration may reduce dependence on CT in selected elective applications [33,73,74,75,76,93]. The clinical objective should not be modality replacement for its own sake, but acquisition of sufficient task-specific geometry with the lowest reasonable burden and the best representation of the tissues relevant to the procedure.

#### 4.6.3. In Silico Testing Before Implant Selection

Patient-specific FE and multibody models could allow surgeons to test several fixation, alignment, graft, or implant scenarios before surgery. The key requirement is calibration against experimental or in vivo reference data. The emerging literature on validated knee contact mechanics, ACL reconstruction, patellofemoral tracking, and OA geometry suggests that this transition is technically plausible [49,50,51,55,56,57,84,85,94,95].

#### 4.6.4. Closed-Loop Surgery

Navigation, robotics, intraoperative sensors, postoperative imaging, and functional data can transform a one-time plan into a feedback system. A closed-loop system should specify update rules, validation after updating, drift monitoring, and clinical accountability. This is a multidimensional combination of execution, outcomes, and adaptivity, not a single maturity level [14,51,54,55,56,57,84,86].

#### 4.6.5. Decision Twins Rather than Anatomy Twins

Anatomical replication alone may not be the most clinically valuable endpoint. The randomized digital-twin decision-aid study in knee osteoarthritis illustrates a broader use: the patient-specific representation can support treatment choice and shared decision-making rather than merely reproduce anatomy [56]. Future orthopedic twins may therefore be judged by decision quality, concordance, and avoided low-value interventions as much as by geometric fidelity.

#### 4.6.6. Provisional I2I Reporting Domains

A practical next step is formal development of the provisional I2I reporting domains. They should undergo stakeholder review, Delphi consensus, pilot application, inter-rater reliability testing, and revision before being described as a reporting statement. Existing guidance remains primary for its own scope: CLAIM for imaging AI, TRIPOD+AI for prediction models, DECIDE-AI for early live AI evaluation, IDEAL/IDEAL-D for surgical and device innovation, ASME/FDA guidance for computational-model credibility, and RSNA guidance for medical 3D printing [10,11,12,13,14,15,16,17,18,29].

Table 5 therefore presents provisional reporting domains rather than a validated checklist. Their proposed contribution is to connect source diagnosis and acquisition with geometry, mechanics, decision relevance, execution, outcomes, implementation, and feedback in one traceable record. Formal content validation and demonstration of reproducible use are future work.

This conceptual synthesis has important limitations. The purposive search and prespecified seed examples may have anchored both the framework structure and the subsequent source mapping; adding the broader literature improves traceability but does not eliminate this selection bias. Several seeds also arise from an overlapping research network, as disclosed in Section 2.2 and Supplementary Table S1. In addition, the absence of a formal study-level risk-of-bias and certainty assessment means that the evidence map cannot establish the strength or generalizability of supporting effects. The framework should therefore be interpreted as a hypothesis-generating organizational model requiring independent systematic evaluation, consensus development, and prospective validation.

## 5. Conclusions

Digital orthopedics is best evaluated as an information pathway rather than a collection of devices. The revised I2I framework adds two clinical checkpoints, treats diagnostic adequacy as a prerequisite, separates measurement and transformation uncertainty from model, decision, implementation, and feedback failures, and replaces the original ordinal maturity ladder with a multidimensional profile. Its contribution is cross-stage traceability across diagnosis, geometry, mechanics, decisions, execution, outcomes, and adaptation. The cited evidence remains heterogeneous and weighted toward technical and preclinical validation. The E0–E8 domains, maturity profile, provisional I2I record, and reporting domains are author-derived and unvalidated; they require consensus development, independent pilot classification, and reliability testing before clinical, regulatory, or reporting use.

## Figures and Tables

**Table 2:** Model validity matrix.

Model	Primary Value	Key Limitation	Preferred Validation Target	Specific Evidence
Imaging and morphometry	Patient-specific anatomy	Resolution and observer effects	ICC or kappa for reliability; bias, limits of agreement, landmark error	[22,45,46]
Segmentation and mesh	Explicit geometry	Boundary and editing bias	Dice plus HD95, surface Dice, ASSD, volume and landmark error	[1,28,46,47]
Anatomical 3D print	Spatial understanding	Print dimensional and material error	Image-to-mesh and mesh-to-print deviation	[1,37,48]
Biomechanical printed surrogate	Controlled comparative testing	Anisotropy, biology and soft-tissue omission	Material calibration and cadaveric or experimental comparison	[2,3,4]
Finite-element or multibody model	Internal mechanics and scenario testing	Constitutive, boundary and loading assumptions	Verification, mesh convergence, sensitivity and independent validation	[12,13,49,50,51]
Patient-specific guide	Direct plan transfer	Fit, registration and operator error	Postoperative plan-to-result deviation and adverse events	[5,30,40,52,53]
Digital twin or adaptive model	Longitudinal prediction	Update logic, drift and validation burden	Prospective recalibration, prediction error and clinical utility	[14,54,55,56,57]

**Table 3:** Provisional E0–E8 uncertainty and failure domains with operational boundaries.

Domain	Conceptual Class	Aligned I2I Stage	Operational Boundary	Recommended Metric or Safeguard
E0	Diagnostic uncertainty	I2I-0	Disease identity or clinical diagnosis before modeling	Reference standard, differential diagnosis, explicit uncertainty
E1	Acquisition uncertainty	I2I-1	Information encoded by the source dataset	Voxel or slice dimensions, SNR, artifact and protocol
E2	Measurement uncertainty	I2I-2	Landmarks, dimensions or phenotypes derived from images	ICC or kappa for reliability; bias, limits and absolute error for agreement
E3	Transformation uncertainty	I2I-3	Segmentation, smoothing, mesh repair or registration	Dice plus surface and landmark metrics relative to tolerance
E4	Fabrication or surrogate uncertainty	I2I-4	Digital-to-physical conversion and material representation	Dimensional QA, material calibration, orientation and repeatability
E5	Model-form and parameter uncertainty	I2I-4 to I2I-5	Constitutive laws, mesh, contacts, loads and calibration	Verification, mesh convergence, sensitivity and independent validation
E6	Decision failure	Early checkpoint and I2I-6	Inappropriate eligibility, indication or plan despite available information	Evidence, alternatives, patient goals and documented rationale
E7	Implementation deviation	I2I-7	Difference between validated plan and achieved execution	Registration logs, seating checks and plan-to-result error
E8	Feedback failure	I2I-8	Absent, biased or unusable outcome data	Postoperative anatomy, PROMs, harms and longitudinal update

**Table 4:** Image-to-Intervention stages and validation questions.

Stage	Name	Core Question	Typical Output	Specific Evidence
I2I-0	Clinical context, diagnosis and early eligibility	Is diagnosis adequate and is advanced modeling justified?	Diagnosis, differential and eligibility decision	[9,19,31]
I2I-1	Image acquisition	Does the dataset contain task-specific information?	DICOM or volumetric imaging	[20,73,75,76]
I2I-2	Quantitative anatomy	Are measurements reproducible and within tolerance?	Angles, areas, volumes and phenotypes	[22,45,77,78]
I2I-3	Digital reconstruction	Does segmentation preserve task-relevant anatomy?	Mask, mesh, STL or STP	[1,21,46,47]
I2I-4	Physical or computational surrogate	Which properties must the surrogate preserve?	3D print, CAD, FE or multibody model	[2,3,49,55]
I2I-5	Verification and validation	Does behavior agree with an appropriate reference?	Error metrics, sensors or experimental comparison	[12,13,42,43,50]
I2I-6	Model-informed decision checkpoint	Does validated information support the indication or technical choice?	Proceed, modify, alternative care or non-intervention	[8,56,58,59]
I2I-7	Execution	How closely was the validated plan reproduced?	Guide, navigation or robot execution error	[30,40,52,66]
I2I-8	Outcome feedback	Did the plan translate into benefit and usable feedback?	Imaging, PROMs, harms, cost and model update	[56,60,86,88]

**Table 5:** Provisional I2I reporting domains for future consensus development.

Domain	Minimum Reportable Item	Relationship to Existing Guidance
Diagnosis and early eligibility	Condition, reference standard, uncertainty and justification for advanced modeling	Extends Fryback–Thornbury decision linkage [9]
Acquisition	Modality, protocol, resolution, artifact and task sufficiency	Complements RSNA and AI reporting guidance [15,18]
Measurement and reconstruction	Landmarks, reliability, agreement, segmentation method and surface metrics	Complements CLAIM and technical QA [15,28]
Physical or computational surrogate	Intended preserved property, manufacturing or model assumptions and context of use	Complements RSNA, ASME and FDA guidance [12,13,18,29]
Decision	Eligibility or technical choice affected, alternatives and appropriateness	Complements IDEAL and DECIDE-AI [10,11,17]
Execution	Transfer method, registration, human factors and plan-to-result accuracy	Adds explicit operative traceability
Outcomes and harms	Postoperative anatomy, PROMs, complications, reoperation, cost and workflow time	Links technical change to patient and system value
Feedback and governance	Update rules, drift, provenance, privacy, cybersecurity and accountability	Complements digital-twin lifecycle guidance [14]

## Data Availability

No new patient-level datasets were generated. The audited citation-to-framework evidence map supporting this conceptual synthesis is provided as Supplementary Table S1; all round-four modifications, including corrected source characteristics and findings, sample or experimental unit, stage mapping, summary counts, verification notes, and seed-provenance disclosures, are marked in blue.

## Supplementary Materials

The following supporting information can be downloaded at: https://www.mdpi.com/article/10.3390/diagnostics16213467/s1, Table S1 Citation and I2I Evidence Map—Round-Four Audit; Citation to I2I evidence map [1,2,3,4,5,6,7,8,9,10,11,12,13,14,15,16,17,18,19,20,21,22,23,24,25,26,27,28,29,30,31,32,33,34,35,36,37,38,39,40,41,42,43,44,45,46,47,48,49,50,51,52,53,54,55,56,57,58,59,60,61,62,63,64,65,66,67,68,69,70,71,72,73,74,75,76,77,78,79,80,81,82,83,84,85,86,87,88,89,90,91,92,93,94,95].

## Abbreviations

The following abbreviations are used in this manuscript:

2D	Two-dimensional
3D	Three-dimensional
ACL	Anterior cruciate ligament
AI	Artificial intelligence
CAD	Computer-aided design
CT	Computed tomography
DIC	Digital image correlation
DICOM	Digital Imaging and Communications in Medicine
E0–E8	Error domains 0–8 of the I2I uncertainty taxonomy
FE	Finite-element
I2I	Image-to-Intervention
IEEE	Institute of Electrical and Electronics Engineers
MEDLINE	Medical Literature Analysis and Retrieval System Online
MPFL	Medial patellofemoral ligament
MRI	Magnetic resonance imaging
OA	Osteoarthritis
PRISMA	Preferred Reporting Items for Systematic Reviews and Meta-Analyses
PROMs	Patient-reported outcome measures
PSI	Patient-specific instrumentation
QA	Quality assurance
RDD	Rosai–Dorfman disease
STL	Stereolithography file format
STP	STEP (Standard for the Exchange of Product model data) file format
TKA	Total knee arthroplasty
ASSD	Average symmetric surface distance
CLAIM	Checklist for Artificial Intelligence in Medical Imaging
DECIDE-AI	Developmental and Exploratory Clinical Investigations of Decision-support systems driven by Artificial Intelligence
HD95	95th-percentile Hausdorff distance
ICC	Intraclass correlation coefficient
IDEAL	Idea, Development, Exploration, Assessment, Long-term follow-up
RSS	Root-sum-square
TRIPOD+AI	Transparent Reporting of a multivariable prediction model for Individual Prognosis Or Diagnosis plus AI
V&V	Verification and validation
